# The Oxidative Drug Combination for Suppressing KRAS G12D Inducible Tumour Growth

**DOI:** 10.1155/2022/9426623

**Published:** 2022-12-30

**Authors:** Dinara Begimbetova, Assiya Kukanova, Fatima Fazyl, Kenzhekyz Manekenova, Talgat Omarov, Agata N. Burska, Medina Khamijan, Alexandr Gulyayev, Bakytgul Yermekbayeva, Abay Makishev, Timur Saliev, Kanat Batyrbekov, Chokan Aitbayev, Zhanat Spatayev, Dos Sarbassov

**Affiliations:** ^1^National Laboratory Astana, Nazarbayev University, Astana, Kazakhstan; ^2^Department of Oncology, Astana Medical University, Astana, Kazakhstan; ^3^Department of Pathological Anatomy, Astana Medical University, Astana, Kazakhstan; ^4^Department of Biology, School of Sciences and Humanities, Nazarbayev University, Astana, Kazakhstan; ^5^S.D. Asfendiyarov Kazakh National Medical University, Almaty, Kazakhstan; ^6^National Research Oncological Centre, Astana, Kazakhstan

## Abstract

**Background:**

Kirsten rat sarcoma (KRAS) protein is an essential contributor to the development of pancreatic ductal adenocarcinoma (PDAC). KRAS G12D and G12V mutant tumours are significant challenges in cancer therapy due to high resistance to the treatment.

**Objective:**

To determine how effective is the ATO/D-VC combination in suppression of PDAC the mouse transgenic model. This study investigated the antitumour effect of a novel combination of arsenic trioxide (ATO) and D-ascorbic acid isomer (D-VC). Such a combination can be used to treat KRAS mutant cancer by inducing catastrophic oxidative stress.

**Methods:**

In this study, we examined the effectiveness of ATO and D-VC on xenograft models—AK192 cells transplanted into mice. Previously, it has been shown that a high concentration of Vitamin C (VC) selectively can kill the cells expressing KRAS.

**Results:**

The results of this study demonstrated that the combination of VC with a low dose of the oxidizing drug ATO led to the enhancement of the therapeutic effect. These findings suggest that the combined treatment using ATO and D-VC is a promising approach to overcome the limitation of drug selectivity and efficacy.

## 1. Introduction

KRAS, a member of the RAS family of protooncogenes, has been associated with a range of high-grade malignancies. KRAS has been identified as the most frequently mutated gene in human cancers that is responsible for 30% of all human carcinomas [1–4]. Studies found that the mutant version of KRAS was presented in 90% of pancreatic cancers [5–7], and up to 40% of colorectal cancers [3]. RAS interacts with proteins involved in many cellular processes, including cell growth, differentiation, mitosis, and metabolism. These interactions lead to the activation of effector proteins such as Raf kinase, PI3K, and Akt, which have been found to have important roles in tumour formation [8, 9]. Inhibition of KRAS signalling has been considered as a promising therapeutic strategy. However, there is no significant progress in the development of specific KRAS inhibitors yet.

The KRAS gene, a protooncogene, encodes a 21-kD guanosine triphosphate (GTP)/guanosine diphosphate (GDP) binding protein. It is involved in the transcriptional regulation of gene products involved in the response to many extracellular stimuli. It is located on the short arm of chromosome 12 and represents the RAS family of oncogenes [10]. KRAS is most commonly mutated in pancreatic cancer (90-95%) where it results in overexpression and activation of KRAS-driven oncogenic signalling that often contributes to tumorigenesis [11]. The KRAS gene codes for a protein found in the tyrosine kinase subunit of the MAPK cascade, which is activated upon binding of IL-1*β* to cause cell death. The classical pathway of cell signalling involves epidermal growth factor receptor (EGFR), hepatocyte growth factor (HGF), and insulin-like growth factor (IGF-I) binding to their respective ligands [12]. KRAS mutations lead to an abnormal function of these effector molecules, which results in uncontrolled cell proliferation, resulting in cancers such as lung adenocarcinoma and pancreatic ductal adenocarcinoma. Among three isoforms, KRAS mutations cause most human cancers, with over 17% of all cancer types [10].

Previous studies have shown that cancer cells with KRAS mutations exhibit rewired glucose metabolism and higher levels of GLUT1 expression. KRAS mutant tumors tend to be more glycolytic than wild-type tumors and require glycolysis for survival and proliferation [13]. Further, GLUT1 overexpression is sufficient to suppress tumor growth in in vitro and in vivo models of KRAS mutant cancers via disrupting redox homeostasis. These results reveal a selective role for GLUT1 in the metabolic drivers of tumor growth and offer a new therapeutic target for treating these tough-to-treat cancers.

Redox homeostasis is an important process that provides both normal and cancer cells with the energy they need to grow and spread. However, malignant cells often show abnormal redox reactions. Therefore, cancer cells are not able to regulate their intracellular levels of reactive oxygen species (ROS) in response to oxidative stress and signals that control growth and differentiation [14]. In this context, altering redox homeostasis is a promising strategy for either preventing or treating cancer. Several lines of evidence demonstrated that excessive or sustained ROS production can be cytotoxic [15]. In particular, hyperproduction of H_2_O_2_ can impair cancer cells development as a result of an increase of endogenous ROS levels or decrease in the cellular glutathione levels.

**Figure 1:**
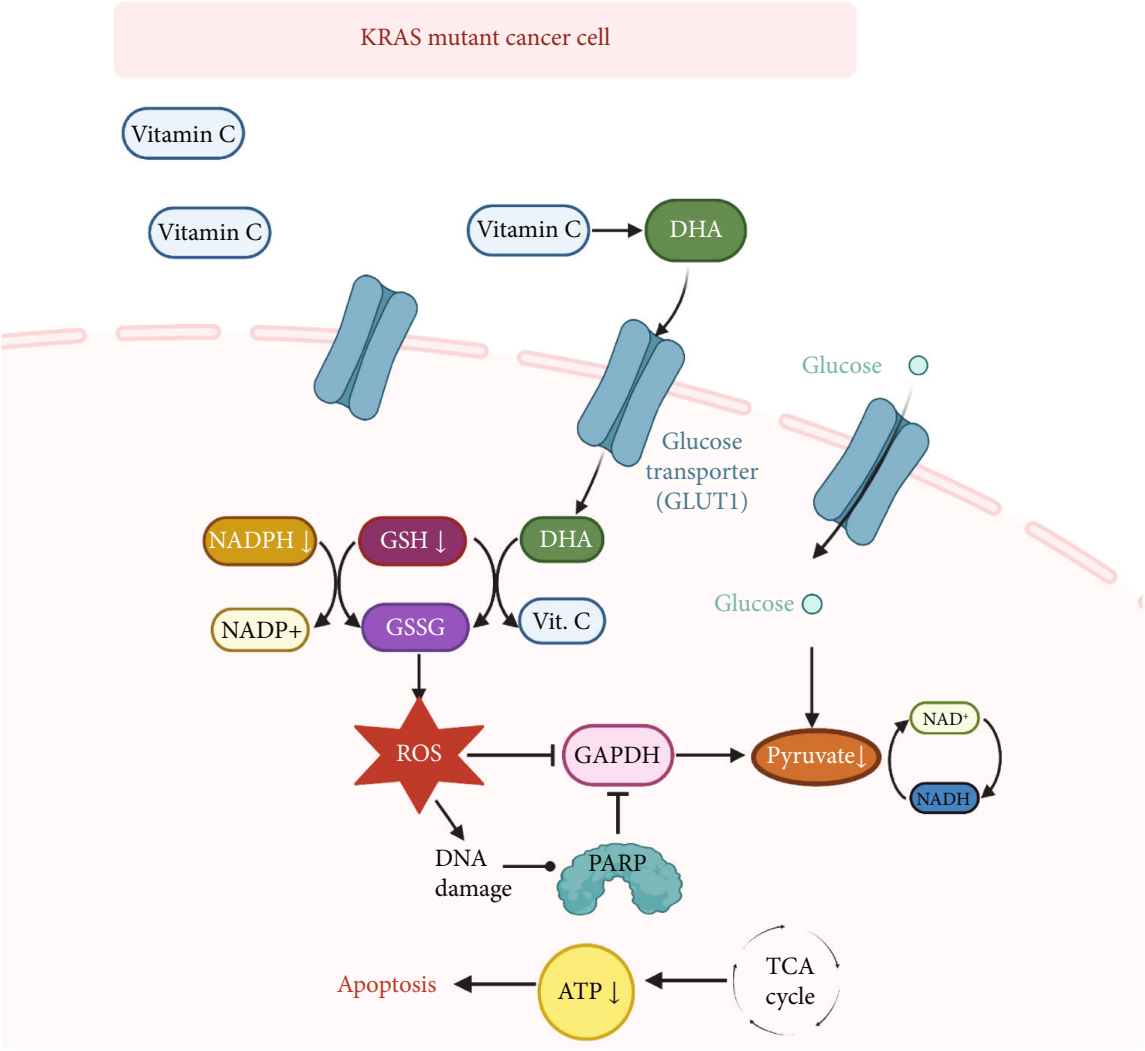
Mechanism of action of VC in KRAS mutant cancer cells. Following the extracellular oxidation of VC to Dehydroascorbic Acid, it is transported into the cell by glucose transporter GLUT1. Inside the cell, DHA is converted back to reduced form at the expense of GSH and NADPH. Depletion of GSH and NADPH increases the ROS generation and DNA damage. Created with http://BioRender.com.

Ascorbic acid functions as an enzymatic cofactor and is the primary antioxidant in vivo, protecting cells from damage by reactive oxygen species such as free radicals. The prooxidant effect of vitamin C with subsequent release of oxygen free radicals at pharmacological/milli-molar concentrations is responsible for the anticancer effect [5]. In humans, vitamin C is necessary for collagen synthesis and the activity of several enzymes involved in the metabolism of amino acids and the biosynthesis of neurotransmitters [16]. Vitamin C (ascorbic acid) is a powerful antioxidant that can donate a hydrogen atom and form a relatively unreactive ascorbate free radical. The first step in the chemical reaction of ascorbic acid is the loss of one electron. This results in an intermediate, ascorbate radical that has a short life of less than one millisecond. Ascorbic acid forms an aldehyde when losing its second electron, which is a very stable form compared to the ascorbate free radical [17]. Vitamin C exists in two forms, L-ascorbic acid and its oxidized form, dehydroascorbic acid. During free radical scavenging, vitamin C donates high-energy electrons to neutralize free radicals, and it is oxidized to dehydroascorbic acid. Both dehydroascorbic acid and ascorbate radical can be eventually reduced back to ascorbic acid. Ascorbic acid can be regenerated from its oxidized form, dehydroascorbic acid (DHA), and is used again for the synthesis of glutathione (GSH) and other thiols or to regenerate oxidized enzyme cofactor molecules. Vitamin C transport is performed by SVCT 1 and SVCT 2 (Sodium-dependent Vitamin C Transporter), which are integral membrane proteins. SVCT 1 and SVCT 2 were previously identified as the specific carriers of ascorbic acid from the intestine [17]. In contrast, dehydroascorbic acid is transported into cells only by glucose transporter protein type (GLUTs). The responsible transporters of dehydroascorbic acid into red blood cells of humans and mice are GLUT1 and GLUT3/4, respectively. The ring form of dehydroascorbic acid and D-Glucose are structurally similar and can therefore be transported by GLUTs. After being transported into the matrix, DHA is converted back to ascorbic acid at the expense of glutathione (GSH), thioredoxin, and NADPH. Previous studies have shown that KRAS mutant cells enhance glucose uptake through the induction of GLUT1 levels [18, 19]. Accordingly, increasing the DHA uptake can disrupt tumor redox homeostasis and kill KRAS mutant cells (Figure 1).

Both VC isomers have the same mechanism of action in provoking oxidative stress. However, xenograft studies' findings suggest that an unnatural form of Vitamin C (D isomer of Vitamin C) produces better anticancer effects than its natural L-Ascorbic acid analog [20]. The rate of VC oxidation to DHA is different for its two forms. Because of its slow oxidation, D-VC is able to promote the prolonged accumulation of DHA in blood circulation and thus, enhances the uptake of DHA by cancer cells. The optimal therapeutic effect has been achieved due to a slow oxidation process (eight times slower compared to L-VC) of VC to Dehydroascorbic acid (DHA). This means that higher amounts of DHA have been selectively taken up from the bloodstream by cancerous cells [20].

Previously, we confirmed the antitumour effect of oxidative drug combination on the HCT116 xenograft mouse model [20]. Here, we expand the series of experiments evaluating and testing the therapeutic efficacy of ATO/D-VC drug combination by subcutaneously transplanting AK192 cells into Nu and NOD Scid mice.

In this paper, we provide evidence that ATO/D-VC combination therapy is effective against KRAS mutant tumour growth.

## 2. Materials and Methods

### 2.1. Cell Culture and Cell Lines

The mouse AK192 cell line was provided from the original sources by Drs. Haoqiang Ying. AK192 cells were cultured in DMEM/F-12 (Dulbecco's Modified Eagle Medium/Nutrient Mixture F-12) (catalogue #DFL13 from Caisson Labs) supplemented with 10% foetal calf serum (FCS), 2 mMl-glutamine, and penicillin (100 units/ml)-streptomycin (100 *μ*g/ml).

For AK192 cells to obtain 80% confluence, 3 million cells were plated into a 150 mm cell culture dish in a 20 ml cell culture medium in combination with 2 g/L of Doxycycline 2 days prior to the xenograft.

For *in vitro* experiments, AK192 cells were seeded at 150000 per well in 6 well plates, left for 24 h to adhere, and subsequently treated with increased dose of ATO from 5 to 30uM or single drug (5 uM ATO, 1 mM D-VC) or combination of drugs (5 uM ATO and 1 mM D-VC). In some experiments cells were pretreated for 30 min with 5 mM NAC before adding ATO and D-VC treatment.

#### 2.1.1. Flow Cytometric Analysis

Adherent AK 192 cells were harvested by gentle trypsinisation at 24 and 48 h (depending on the experiment), counted using MultiSizer (Beckman). The cells were then resuspended at 1 × 10^6^ cells/ml and stained with propidium iodide (PI) (1ul for 5 min in the dark) to assess % of dead cells and MitoSOX (2.5 nM for 30 min and 37°C) for mitochondrial superoxide production (both from ThermoFisher).

To assess induction of apoptosis by combination of oxidative drugs cells were trypsinsed and combined with collected DMEM to account also for floating apoptotic cells. Suspension was washed once with PBS and then with 1X annexin binding buffer (ABB) and 100*μ*l of 1 × 10^6^ cells/ml were stained with 3ul of AnnexinV-FITC and 1 ul of PI for 15 min at RT in the dark. Afterwards 400 ul of buffer was added and cells were kept on ice until analysis.

Samples were acquired using *Attune* NxT *Flow Cytometer* (ThermoFisher) for all 3 florescent staining according to the manufacturer's instructions. For PI and apoptosis assays 30.000 events were acquired and 10.000 for MitoSOX.

### 2.2. Drug Preparations

The Ascorbic Acid (VC, catalogue #A7506), d-(-) isoascorbic acid (D-VC, catalogue #856061), and Arsenic Trioxide (ATO, catalogue #A1010) used in our study was obtained from Sigma. To prepare 340 mM stock solution of VC, 30 g of D-VC was dissolved in sterile 400 ml PBS solution following the addition of 39 g of sodium bicarbonate (NaHCO3, catalogue #S5761). The solution was constantly stirred for another 5 minutes. Sodium bicarbonate was used as a buffer to bring the acidic (2.3) pH of VC up to physiological levels (pH 7.35) for injection into laboratory animals. The final D-VC solution was adjusted to 500 ml by adding PBS. The D-VC solution was filtered, aliquoted, and kept in a -20°C refrigerator to ensure that the drug was as pure and effective as possible. The stock solution of 330 mM ATO was prepared by mixing and dissolving 13 g of ATO in 1 N sodium hydroxide (NaOH). Then, the total volume of ATO was adjusted to 200 ml. The final solution was filtered and stored in a -20°C refrigerator.

### 2.3. Xenograft and Histological Analysis

The animal studies were performed according to the guidelines provided by National Laboratory Astana. Xenograft studies were conducted to investigate the growth of human Pancreatic ductal adenocarcinoma (PDAC) xenografts in Nu and NOD Scid strains of a mouse obtained from the Institute of Cytology and Genetics, Novosibirsk. A mixture of 2×106 AK 192 cells and Matrigel (catalogue #354248) were injected subcutaneously under isoflurane anaesthesia. Tumour growth was monitored every 2 days with a flexible ruler. Body weight was measured once a week during the experimental period. The drug injections started after 10 days when the tumours reached a size of >0.6 cm in diameter. The animals were treated with a combination of ATO/D-VC at 6 mg/kg and 1.5 g/kg for 2 weeks, respectively. Each group that included xenografts also contained animals that received PBS instead of drugs as a negative control. Mice were sacrificed at 2 weeks' postinjection. The xenograft tissues were examined histologically and quantitatively (weight) by standard protocol. Formalin-fixed, paraffin-embedded cancer tissues were stained with hematoxylin and eosin. Histological analysis was done by microscope Olympus CX41 with the morphometric program.

### 2.4. Statistical Analysis

Statistical significance was determined using GraphPad Prism 9 (Graph Pad Software, San Diego, CA). Data were considered to be significant when p values are <0.05.

## 3. Results

### 3.1. Effect of Increased ATO Concentration on AK 192 Cells

**Figure 2:**
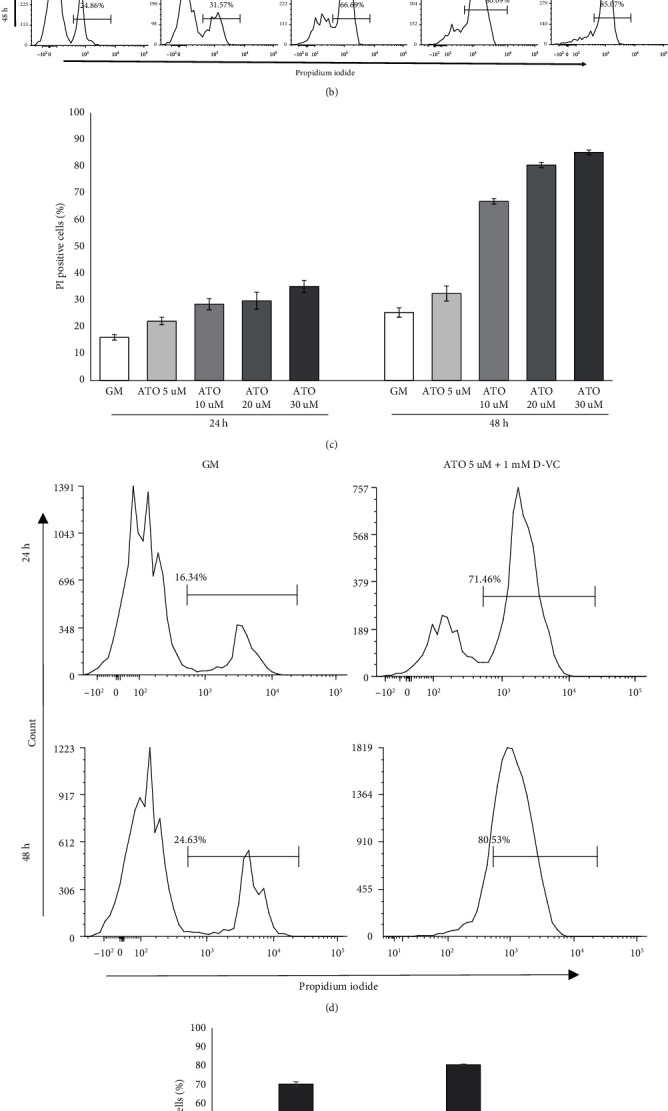
ATO titration and cell death in AK 192 cell line. (a) Morphological changes in AK 192 cells after 24 h of increased dose of ATO. (b) Representative histograms of % of PI positive cells in control (GM) and after treatment with increased dose of ATO at 24 and 48 h. (c) Bar chart of % of dead cells in each condition at 24 and 48 h. (d) Histograms of % of PI positive cells in GM after combined treatment with ATO 5uM and D-VC 1 mM. (e) Bar chart of % of dead cells in GM and after combined treatment with ATO 5*μ*M and D-VC 1 mM at 24 and 48 h.

Ak192 cells were treated with increased dose of ATO from 5 to 30*μ*M. Slowed growth was observed at ATO 10*μ*M and significant morphological changes at 20 and 30*μ*M ATO (Figure 2(a)). Dead cell count was performed by PI staining and flow cytometric analysis. PI intercalates between the bases with a stoichiometry of one molecule of dye per 4-5 base pairs of DNA. It is excluded by viable cells but can penetrate cell membranes of dying or dead cells and in these cells gives strong red-fluorescence signal from stained nuclei. In the first 24 h, ATO treatment slightly increased death rate with increasing concentration however, the increase was not dramatic. While 48 h incubation with drug caused significant death at ATO 10 mM and above (% of PI positive cells above 66%, 80%, and 85%, respectively, for AT0 10,20 and 30 mM) (Figures 2(b) and 2(c)). Only high doses of ATO and prolonged incubation time was able to induce substantial death of AK192 cells; while combination of 5*μ*M ATO and 1 mMD-VC induced death in 70% of AK 192 cells at 24 h and 80% at 48 h. This indicate that ATO/D-VC combination is more effective to induce cell apoptosis than ATO alone, combination also allows for lower dose of ATO being used to induce the cytotoxic effect.

### 3.2. ATO/D-VC Treatment Combination Induces Apoptosis in AK 192 Cell Line

**Figure 3:**
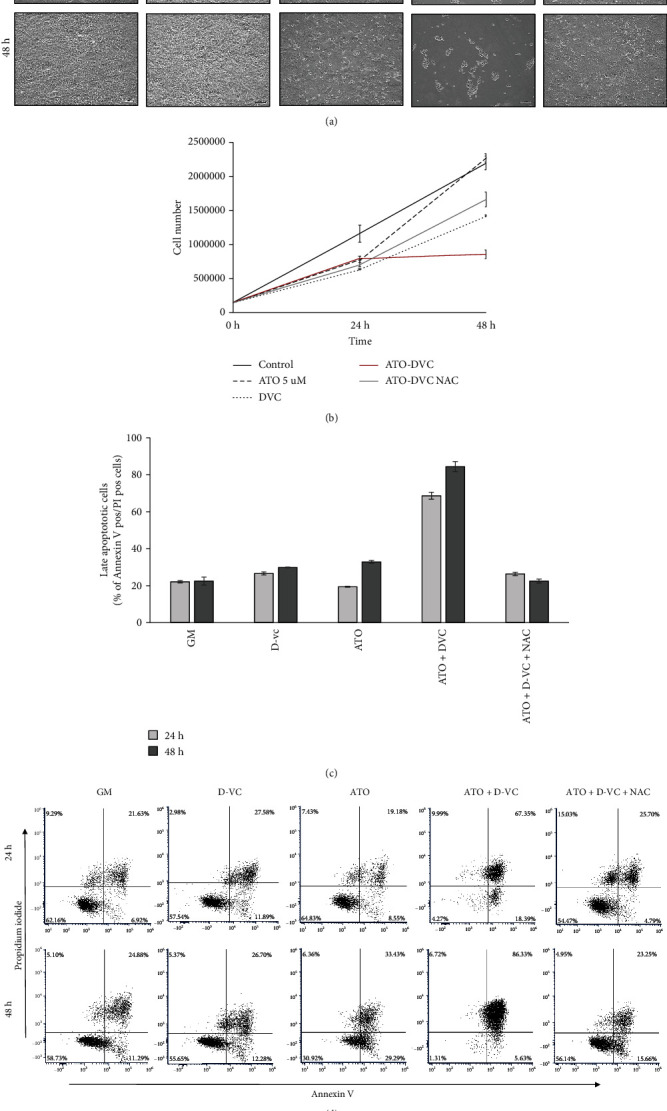
Induction of apoptosis by ATO and D-VC combination in AK 192 KRAS mutant cancer cells. (a) The bright field images of cells after treatment with PBS (GM control), 1 mM D-VC, 5 *μ*M ATO, combination of 1 mM DVC and 5 *μ*M ATO and 1 mM DVC and 5 uM ATO and 5 mM NAC. Images taken at 24 hours top row and 48 h bottom row. Scale bar 50*μ*M. (b) Total cell number of AK 192 cells under different conditions over time. (c) The graphical presentation of the PI positive cell population shown. (d) The apoptotic assay using Annexin-V and PI staining and flow cytometric analysis. The dot plots of the cells described in (a), gate was set using unstained sample.

To further clarify, the additive effect of ATO and D-VC cells were treated with each of the drugs alone and combination and apoptosis was evaluated using Annexin-V and FITC. Morphological changes in AK 192 KRAS mutant cancer cells were observed after treatment with oxidative drugs combination 1 mM DVC and 5 *μ*M ATO but not with ATO or D-VC alone at any of the observed time points. AK 192 cells were also preincubated for 30 min with 5 mM NAC before addition of oxidative drug combination. NAC has ROS scavenging properties and acts directly via the redox potential of thiols, or secondarily via increasing glutathione levels in the cells. Preincubation with NAC blocked effect of ATO and D-VC combination and microscopically good recue effect was observed (Figure 3(a)). Total cell number of AK 192 cells under different conditions were counted using Multisizer. Significantly, reduced cell counts were seen in ATO and DVC treated cells with NAC and ATO and D-VC combination showing higher cell count at 48 h confirming NAC protective effect against oxidative drugs combination (Figure 3(b)). Flow cytometric analysis of apoptotic cells using dual staining with Annexin V and PI indicated significant induction of apoptosis seen as increased % of double positive cells (Annexin V+/PI+). Approximately 70% and 90% of apoptotic cells were observed after combined 5 *μ*M ATO/1 mM D-VC treatment at 24 and 48 h, respectively (Figures 3(c) and 3(d)). Cells preincubated with NAC before ATO/D-VC treatment showed significantly reduced % of apoptotic cells 25 and 23% at 24 and 48 hours indicating protective effect against oxidative drugs combination.

### 3.3. Mitochondrial ROS (mtROS) Production in Response to ATO/D-VC Treatment Combination in AK 192 Cell Line

**Figure 4:**
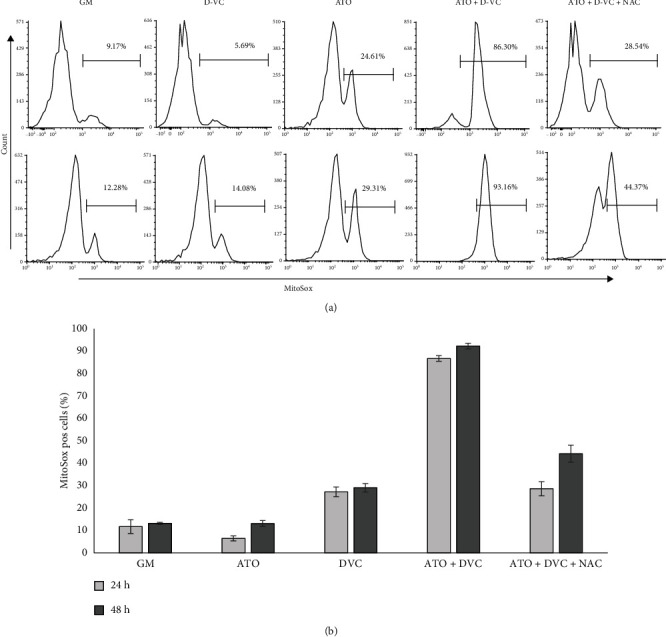
Flow cytometric analysis of mitochondrial ROS production in AK 192 cells under treatment conditions. (a) Histograms with % of MitoSox positive cells (gate set up on unstained sample) for conditions treated with PBS (GM), 5*μ*M ATO, 1 mM D-VC, combination of 5uM ATO and 1 mM D-VC, combination of 5uM ATO and 1 mM D-VC and 5 mM NAC A. (b) analysis of MitoSox was done in triplicates and the average values are show on a bar chart.

To confirm that ATO and DVC combination of drugs is cytotoxic via induction of ROS, mtROS production was assessed by flow cytometry using MitoSox probe which detects superoxide production. In comparison to control (GM), treatment with ATO/D-VC induced 7.3 and 7 fold increase in mtROS at 24 and 48 h, respectively (Figures 4(a) and 4(b)). Preincubation of cells with 5 mM NAC for 30 min before ATO/D-VC treatment reduced significantly mtROS production by 3 and 2 times at 24 and 48 h indicating that blocking ROS production helps AK 192 cells to survive treatment with oxidative drugs combination.

### 3.4. ATO/D-VC Combination Is Effective in Suppression of KRAS Inducible Tumour Growth

**Figure 5:**
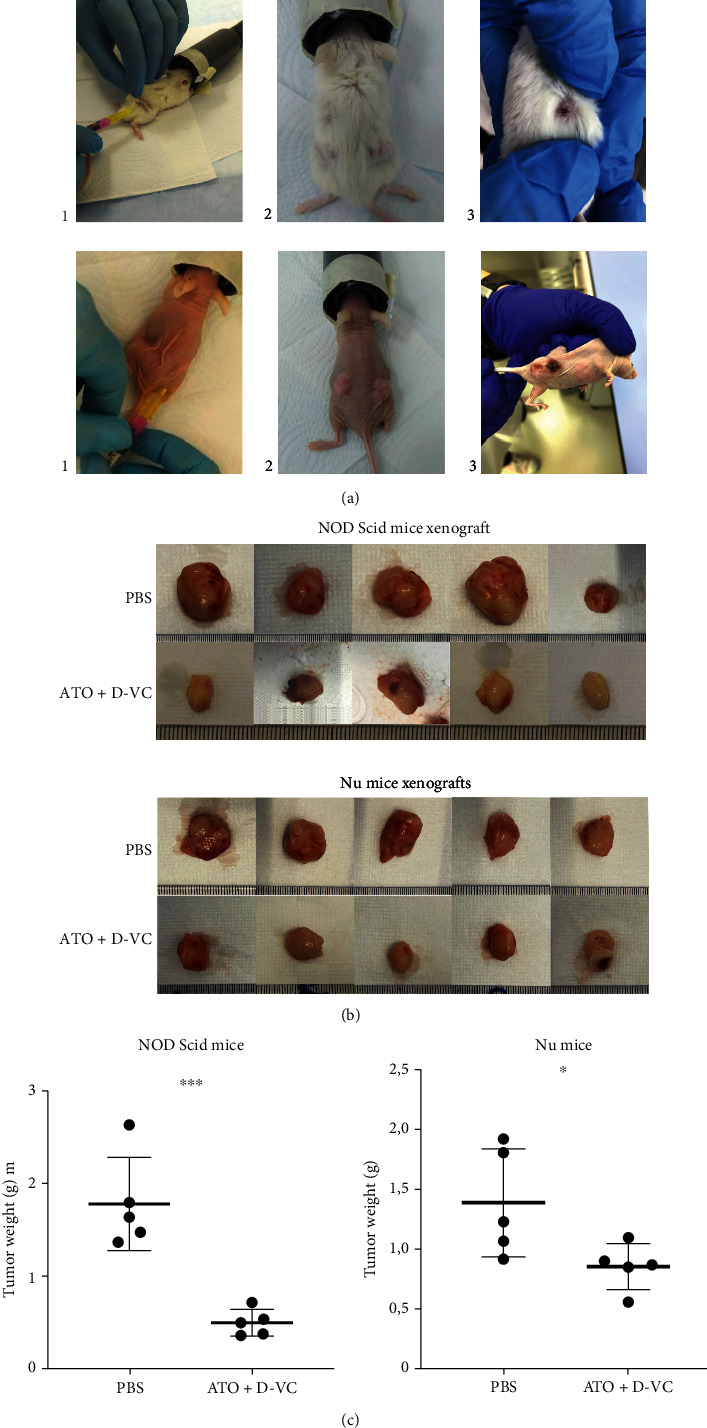
Xenografts. (a) Transplantation of AK 192 cancer cells into NOD Scid and Nu mice. (b) Representative image of dissected tumor. (c) Experimental group had reduced tumor weight at sacrifice.

KRAS mutated pancreatic cancer is characterized by high KRAS expression and the presence of a chirality-dependent tumour microenvironment. The action of VC is sensitive to chirality and thus the activity of ATO can be selectively potentiated when combined with VC at low doses. Considering that the ATO/VC combination is effective in cell culture, we tested it in a xenograft mouse cancer model. AK 192 cancer cells were transplanted into NOD Scid and Nu mice subcutaneously and the drugs were injected after 10 days when the tumours reached a size of >0.6 cm in diameter (Figure 5(a)). The combination of ATO and D-VC was administered as two consecutive intraperitoneal infusions. Four groups were studied as follows: control group NOD Scid and Nu (no treatment), ATO/D-VC NOD Scid and Nu groups. Two hours after the administration of ATO, the VC was injected. In order to maximize its efficacy, we used the 2 h interval between the ATO and VC injections.

To avoid fluctuations in the blood glucose level, mice were fasted two hours before receiving the injections of ATO or VC and were fed 2 hours after the administration of D-VC.

The dose of VC was 1.5 g/kg. Within the second week of injections, we observed tumour shrinkage in mice that were treated with an ATO/D-VC combination (Figure 5(b)). During the treatment, the tumour capsule, which at the beginning of the treatment had a rounded whole shape, was crushed and became flattened.

We employed two strains of mice: NOD Scid and Nu. After the 15th (last) injection of the drug, analysis of the tumour by weight showed that the combination of ATO/D-VC caused potent tumour shrinkage in NOD Scid mice; tumour shrinkage was slightly less in Nu mice (Figure 5(b)).

The results of the study showed a statistically significant reduction in tumour weight in both types of ATO/D-VC injected animals (NOD Scid and Nu) compared to the negative PBS control, *p* ≤ 0.0006, *p* ≤ 0.004, *p* ≤ 0.0404, and *p* ≤ 0.04, respectively (Figure 5(c)).

ATO/D-VC has been shown an effective in suppressing tumour growth of the KRAS G12D mutant PDAC tumours.

### 3.5. Effect of ATO/D-VC on NOD Scid and Nu Xenograft Mice by Histological Analysis

#### 3.5.1. ATO/D-VC Combination Is Effective in Suppression of KRAS Inducible Tumour Growth in NOD Scid Xenograft Mice

**Figure 6:**
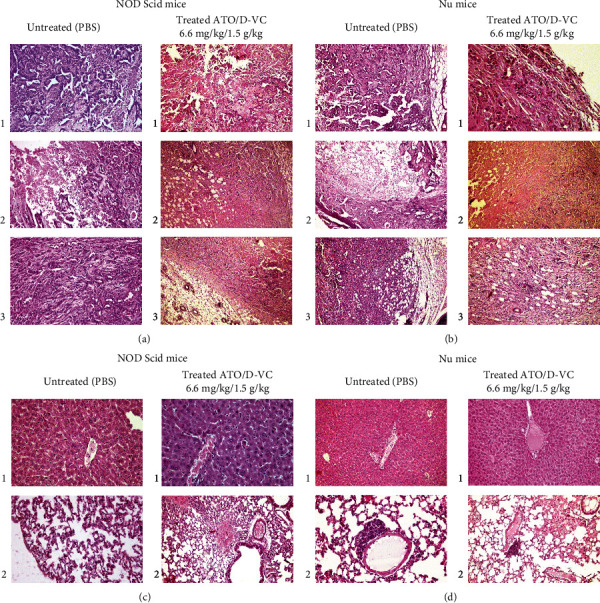
Histological analysis. The hematoxylin and eosin stained tissue, ×200. (a) Histological analysis of tumour xenografts from immune-deficient NOD Scid mice with and without ATO/D-VC treatment. Untreated (PBS control) are as follows: (1) glandular structures with papillary hyperplasia of the epithelium, (2) inflammatory infiltration, and (3) oedema of the stroma. Treated ATO/D-VC: 1- necrosis. 2- subtotal necrosis. 3 - subtotal necrosis. (b) Histological analysis of tumour xenografts of immune-deficient Nu mice with and without ATO/D-VC treatment. Untreated (PBS control): 1- Tumour invasion into the capsule. 2 - The glands are separated by proliferating connective tissue. Treated ATO/D-VC: 1 - sclerosis and lipomatosis of the tumour. 2 - tumour necrosis. 3 - Decrease in growth index. (c) Histological analysis of organs of NOD Scid xenograft mice with and without ATO/D-VC treatment. Untreated (PBS control): 1 - Liver. The hepatic lobules retain a beam structure. Moderate Kupffer cell hyperplasia. 2 - Lungs. Moderate swelling of the interalveolar septa. Bronchi: the focal proliferation of the epithelium, partial desquamation, secretory active epithelium. Treated ATO/D-VC are as follows: (1) liver: Irregular hyperplasia of Kupffer cells. Small foci of extramedullary hematopoiesis are found in apoptotic cells. Fragmentation of the nucleus, pycnosis of the nucleus. Cytolysis of individual hepatocytes is noted. In the subcapsular zones, areas with signs of vacuolar dystrophy of hepatocytes are determined in places. (2) Lungs.: Slight focal alveolar oedema. (d) Histological analysis of organs of Nu xenograft mice with and without ATO/D-VC treatment. Untreated (PBS control) is as follows: (1) liver: moderate hyperplasia of single Kupffer cells. Cytolysis of single hepatocytes. Periportally moderate oedema in the liver. (2) Lungs: single perivascular lymphocytic infiltrates. Treated ATO/D-VC is as follows: (1) liver: extramedullary hematopoiesis. (blast cells). Moderate hyperplasia of Kupffer cells, cytolysis of certain groups of hepatocytes. (2) Lungs: single perivascular lymphocytic infiltrates.

In NOD Scid mice treated with ATO/D-VC, the tumour node within the subcutaneous soft tissues was examined histologically and confirmed to be in a state of total necrosis meaning cells had been destroyed by inflammation (Figure 6(a)). The tumour tissue is surrounded by bundles of muscle fibres. Inflammatory reactions are present on the periphery of the tumour tissue. Only the contours of tumour cells are identified.

A productive inflammation on the periphery of a necrotic tumour node and adipose tissue inflammation was observed. Tumour tissue is represented by slit-like cavities and short strands of tumour cells (subtotal necrosis).

Pseudobulbar and glandular structures remained intact in subtotal necrosis. Tumour tissue was with desmoplastic changes (subtotal necrosis with glandular structures).

Histological analysis of nontreated NOD Scid xenograft mice tumour, revealed the nodular formation that is surrounded in places by a thin fibrous capsule with extensive areas of necrosis and focal haemorrhages (Figure 6(a)). Under the capsule, strands of muscle cells with dystrophic changes are determined, and the same cells are determined in places in the tumour stroma.

The tumour tissue was preserved on the periphery of the node and represented by glandular, pseudo-tubular and nested structures, consisting of epithelial cells with oxyphilic cytoplasm and polymorphic nuclei with an uneven distribution of chromatin. In places, the pseudo tubular structures were unevenly expanded. The presence of papillae and cribriform structures was observed. Mitoses take place. In tumour epithelial cells, areas of cytolysis and polymorphism were observed.

In the zone of tumour tissue necrosis, fibroblastic changes were noted in the form of sclerosis of the papillary stroma and atrophy of tubular structures. Stromal oedema, stasis of erythrocytes in micro vessels, and scattered lymphocytic reaction are present on the periphery of the tumour. We observed small diapedetic haemorrhages. In addition, we found stasis of erythrocytes with an admixture of leukocytes in the lumen of the vessels. Results demonstrated the leukocyte infiltration around the necrosis area. There is an accumulation of secretion with an admixture of leukocytes (inflammatory infiltration) in the lumen of some glands. Stromal oedema, muscle cells in a state of myolysis, and atrophy in the thickness of the tumour tissue were also detected.

#### 3.5.2. ATO/D-VC Combination Is Effective in Suppression of KRAS Inducible Tumour Growth in nu Xenograft Mice

Histological analysis showed the signs of pronounced fibrosis in the stroma (Figure 6(b)). The nodule was surrounded by fatty tissue in a thin fibrous capsule, pseudobulbar structures, pseudoglandular structures, and glands dilated with papillary structures (solid tumour with areas of microsolid structure, and decreased tumour differentiation). The expressed oedematous stroma and tumour lipomatosis was observed.

In Nu ATO/D-VC, the tumour capsule invasion was detected. The glands were separated by proliferating connective tissue. In tumour necrosis with sclerosis, in places, the zone of necrosis reaches the capsule. The analysis showed tumour necrosis, discrete tumour cells under the capsule, inflammation, extensive subcapsular fibrosis, capsule invasion, sclerosis, and lipomatosis of the tumour.

The signs of necrosis and shrinkage of tumour indicated that ATO/D-VC is effective in reducing tumour of both NOD Scid and Nu mice. Thus, we demonstrated that the unnatural enantiomer of VC (D-VC) in combination with ATO is effective in suppressing the KRAS mutant tumour.

The results showed that ATO/D-VC is capable of inhibiting tumour growth of the KRAS G12D mutant PDAC cancers. ATO/D-VC was effective in reducing tumour weight of both NOD Scid and Nu mice.

#### 3.5.3. The ATO/D-VC Oxidation Drug Combination Is Not Toxic in Mice

**Figure 7:**
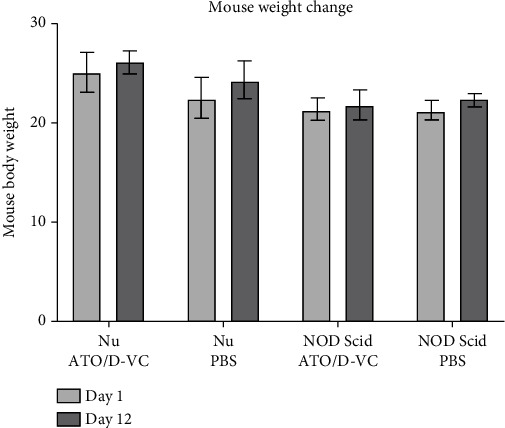
Mice weight change. The difference in weight change between ATO/D-VC treated and untreated mice was not statistically significant. All mice did not lose weight.

Histological analysis of mouse organs (liver, kidney, lungs, and cardiac tissue) did not detect significant abnormalities or signs of necrosis (Figures 6(c)–6(d)). The difference in weight change between ATO/D-VC treated and untreated mice was not statistically significant. No weight reduction was observed in any of the groups (Figure 7).

We noticed the effect of xenograft tumour on the studied organs of mice. These processes are a normal immune response to the presence of a tumour.

## 4. Discussion and Conclusion

Our findings suggest that the combination of ATO and VC can lead to oxidative stress-mediated KRAS mutant cancer cell death. A tumour analysis revealed that the average weight of tumour of ATO/D-VC treated NOD Scid and Nu mice were lower by 72% (3.57 times) and 38% (1.62 times) compared to the control group, respectively (Figure 5(c)).

At the beginning of the injection, the implanted tumor under the skin of the animals was a uniform round structure with a diameter of 0.6 cm. On the 9th day, in the experimental group of animals treated with a combination of oxidizing drugs ATO/D-VC, macroscopic examination showed primary signs of a decrease in tumour volume compared to animals without treatment. In the following days, these signs intensified and the tumor took on a flattened shape. At the end of the experiment, depletion of the tumour was observed, followed by the scabbing process.

The exact mechanism of how ROS production triggers apoptosis is still unclear but there is an evidence of suicidal ROS production by mitochondria (SRPM) under oxidative stress [19]. The metabolic shift of higher glucose consumption in malignant cancer cells makes them more susceptible to the oxidation.

Chronic oxidative stress has been considered as a factor of cancer-specific vulnerability, as well as an adaptive strategy for cancer cells under hypoxic conditions. As a concept, it is based on the redox biology paradigm. The idea is that increased reactive oxygen species generation plays a major role in tumourigenesis. Therefore, dysregulation of the redox system represents a promising target to prevent tumour growth and progression. Despite the therapeutic potential, many current conventional therapies lack a selectivity. Up to date, researchers have developed an inhibitor that selectively induces oxidative stress in tumours while leaving healthy cells unharmed. Here, we tested a novel combination of FDA-approved drugs that have already been considered safe for clinical use.

Arsenic trioxide is a standard chemotherapy agent for treating solid cancers and leukaemia. It kills cancer cells via chemically-induced apoptosis—excess generation of reactive oxygen species (ROS)—which in turn cause intracellular glutathione levels to go down. Cancer cells that show a high level of glutathione (GSH) are more resistant to arsenic trioxide. Researchers have found that cancer cells can be killed by combining arsenic trioxide with agents that deplete intracellular GSH levels such as Vitamin C [21].

ATO and D-VC in combination induce catastrophic oxidative stress and death of cells expressing mutant KRAS in vivo. Mice xenografted with the KRAS G12D oncogene responded well to a drug combination consisting of ATO and D-VC. Moreover, this study found that the ATO/D-VC combination treatment has better efficacy in inhibiting tumour growth in the AK192 xenograft model than the HCT116 cell line transplanted xenograft (average tumour weight was lower by 3.57 and 3.4 times lower compared to the control group, respectively) [20].

A synergistic cytotoxic impact induced by the ATO and D-VC combination indicates that surpassing an oxidative threshold in KRAS mutant cancer cells affects the redox-sensitive cellular systems including the most vulnerable mitochondrial oxidative phosphorylation system (OXPHOS) complexes. Taking it into account, a synergy of the ATO and VC combination in killing KRAS mutant cancer cells might take place by the two step actions. In the first step, a depletion of GSH is mediated by a high dose of VC that disarms a cellular antioxidative shield. In the second step, ATO acts in a full capacity by attacking thiol-reactive groups on cellular proteins with a preference of attacking mitochondrial OXPHOS complexes. It is likely that a direct oxidation of thiol-reactive proteins within OXPHOS complexes leads to uncontrolled ROS production or suicidal mitochondrial ROS production.

To summarize, the proposed combination of drugs could effectively inhibit KRAS-mutant cell growth by generating high concentrations of reactive oxygen species that induce apoptosis and suppress angiogenesis.

A novel anticancer drug combination has been found to be effective in promoting tumour cell death in vivo. These findings indicate the therapeutic potential for the development of new type of anticancer therapy. More studies are needed to prove clinical relevance, efficacy, and safety of the proposed method.

## Data Availability

The data used to support the findings of this study are available upon request from the corresponding authors.
